# Development of highly faceted reduced graphene oxide-coated copper oxide and copper nanoparticles on a copper foil surface

**DOI:** 10.3762/bjnano.7.93

**Published:** 2016-07-11

**Authors:** Rebeca Ortega-Amaya, Yasuhiro Matsumoto, Andrés M Espinoza-Rivas, Manuel A Pérez-Guzmán, Mauricio Ortega-López

**Affiliations:** 1 SEES, Electrical Engineering Department, Centro de Investigación y de Estudios Avanzados del IPN. Av. IPN 2508, Col. San Pedro Zacatenco, Mexico City 07360, Mexico; 2 Nanoscience and Nanotechnology Program. Centro de Investigación y de Estudios Avanzados del IPN. Av. IPN 2508, Col. San Pedro Zacatenco, Mexico City 07360, Mexico

**Keywords:** copper, copper(II) oxide, core–shell, reduced graphene oxide

## Abstract

This work describes the formation of reduced graphene oxide-coated copper oxide and copper nanoparticles (rGO-Cu_2_ONPs, rGO-CuNPs) on the surface of a copper foil supporting graphene oxide (GO) at annealing temperatures of 200–1000 °C, under an Ar atmosphere. These hybrid nanostructures were developed from bare copper oxide nanoparticles which grew at an annealing temperature of 80 °C under nitrogen flux. The predominant phase as well as the particle size and shape strongly depend on the process temperature. Characterization with transmission electron microscopy and scanning electron microscopy indicates that Cu or Cu_2_O nanoparticles take rGO sheets from the rGO network to form core–shell Cu–rGO or Cu_2_O–rGO nanostructures. It is noted that such ones increase in size from 5 to 800 nm as the annealing temperature increases in the 200–1000 °C range. At 1000 °C, Cu nanoparticles develop a highly faceted morphology, displaying arm-like carbon nanorods that originate from different facets of the copper crystal structure.

## Introduction

In the last years, graphene oxide (GO) and reduced graphene oxide (rGO) have emerged as suitable candidates to prepare graphene-based nanocomposites [1–2], including those based on GO/inorganic nanoparticles [3]. The opportunity to combine GO with inorganic nanoparticles has led to nanocomposites with multiple functionalities [4–5]. Because of this, nanocomposites based on GO/inorganic nanoparticles are currently of interest for applications in biomedicine, energy production, electronics and environmental remediation [6–8].

A special class of composites based on GO/inorganic nanoparticles is the one in which the inorganic nanoparticle is protected against degradation by coating with a suitable material (shell) [9–10]. Actually, silica, polymers and graphitic materials hold promise to produce highly stable well-protected metal or metal oxide nanoparticles [11]. In particular, rGO–Cu core–shell nanostructures have been synthesized by CVD [12–13], hydrothermal synthesis [14] and pyrolysis of an organocopper compound [15–17].

In a previous work, the authors reported the effective reduction of chemically exfoliated GO using a copper foil as the GO support. It was found that the final product consisted of rGO sheets decorated with Cu or Cu_2_O nanoparticles. To explain the presence of unoxidized Cu nanoparticles it was suggested that rGO sheets might coat Cu to prevent oxidation [18].

The present work reports further studies on the abovementioned Cu-based nanoparticles. Interestingly, they develop a highly faceted morphology and are actually coated with rGO sheets. The rGO-coated Cu-based nanoparticles developed at temperatures in the range of 200–1000 °C starting from bare Cu_2_O nanocrystals that formed at 80 °C. Their morphology and the inorganic predominant phase strongly depended on the annealing temperature.

## Experimental

### Materials

Graphite flakes (+100 mesh), sodium nitrate (≥99%), potassium permanganate (≥99%) and hexane (≥99%) were purchased from Sigma-Aldrich. Sulfuric acid (95–98%) was obtained from Reproquifin. Hydrogen peroxide (30%), acetone (99.77%) and copper foil (99.99%) were obtained from J.T. Baker. Ethanol (99.5%) was purchased from Reasol. All reagents were used as received without further purification.

### Preparation of the rGO sheets and copper-based nanoparticles composite

As described in our earlier work [18], GO was prepared from natural graphite flakes by a modified Hummers method [19–20]. GO was dispersed in water (2 mg/mL) and then drop cast on a previously cleaned Cu foil (0.02 mm thick and 1 × 1 cm^2^) to form a thin film. Before the high temperature process, the sample was dried at 80 °C under nitrogen flux for 15 min.

The thermal process leading to the reduction of GO and the formation of Cu-based nanoparticles was carried out in an atmospheric pressure CVD apparatus equipped with a quartz tube. The annealing process was performed at 200, 400, 600, 800 and 1000 °C under an inert Ar atmosphere for 1 h. For characterization, the rGO films were peeled off with stainless steel tweezers.

### Characterization

The structure and morphology were studied using transmission electron microscopy (TEM, JEOL 2010 and HR-TEM, JEOL ARM 200F), field emission scanning electron microscopy (FE-SEM, Zeiss Auriga). Energy dispersive X-ray spectrometry (EDS, Bruker Xflash 5010 detector) was used to characterize composition.

## Results and Discussion

### Formation of initial nanoparticles

**Figure 1:**
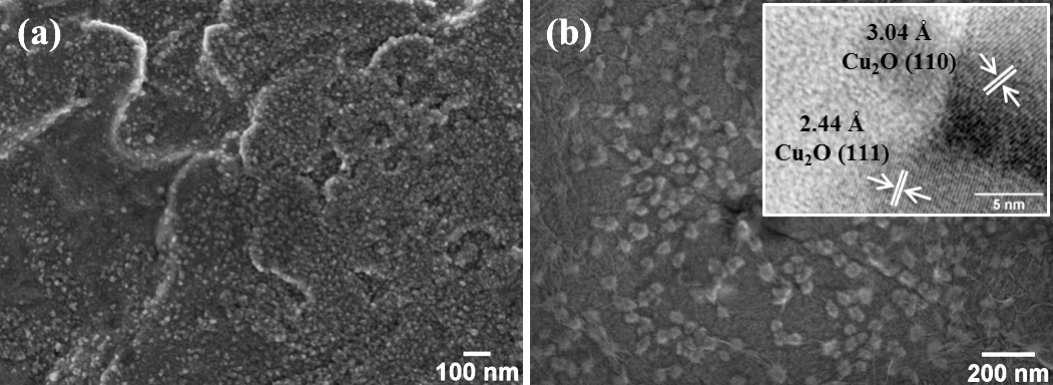
FE-SEM images of the copper foil (a) before and (b) after GO was deposited and dried at 80 °C. The inset of (b) shows the TEM image with an inter-planar spacing of 3.04 and 2.44 Å, corresponding to the (110) and (111) planes of cubic Cu_2_O, respectively.

We prepared a series of samples of GO supported on a Cu foil, as described in the Experimental section. FE-SEM images in Figure 1 show the surface morphology of the substrate copper foil (a) before and (b) after GO was deposited and dried at 80 °C. The bare Cu foil surface exhibits particles 7–8 nm in size and structural defects. The surface chemical composition determined by EDS revealed the existence of oxygen probably forming a native copper oxide layer (Cu*_x_*O) and carbonaceous species as adsorbed impurities. The inset of Figure 1b shows a TEM image of nanoparticles grown at 80 °C.

After being detached and analyzed by TEM, it was seen that well-crystallized Cu_2_O nanoparticles developed during the annealing at 80 °C (inset in Figure 1b). In our previous work we assumed that they developed following a mechanism similar to that proposed by Glover and co-workers [21]. They demonstrated that Cu nanoparticles (CuNPs) can be formed on the surface of copper objects exposed to ambient humidity for a few minutes. We believe that during the drying process similar conditions to those proposed by Glover et al. could be established which lead to the formation of CuNPs, which oxidized further due to interaction with water vapor.

### Development and phase evolution of Cu-based nanoparticles

After being annealed at temperatures of 200–1000 °C under inert atmosphere, both the morphology and phase composition of initial Cu_2_O nanoparticles dramatically changed. In the entire annealing temperature range, faceted rGO-coated copper oxide or copper nanoparticles (rGO-Cu_2_ONPs, rGO-CuNPs) were obtained; the predominant phase of inorganic core being determined by annealing temperature.

**Figure 2:**
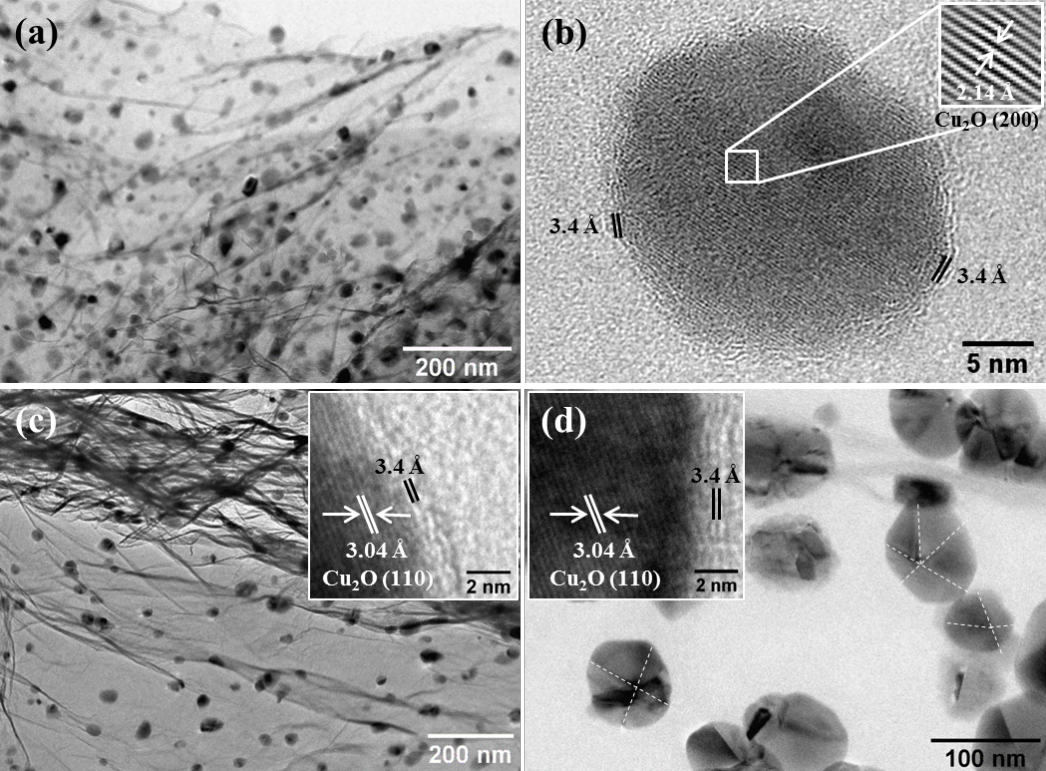
TEM images of samples annealed at (a,b) 200 °C, (c) 400 °C, (d) 600 °C for 1 h. The insets of (b–d) provide details of the interplanar spacing of both, the Cu_2_O core and the rGO shell. The dotted lines on the nanoparticles image of (d) were added to visualize the faceted morphology of the Cu_2_ONPs.

Figure 2 displays TEM images of samples prepared at 200–600 °C for 1 h. In this temperature range, the initial Cu_2_O phase preserves in the inorganic core of rGO-coated NPs. Figure 2b and insets in Figure 2c,d provide details about the interplanar distance of the carbon shell as well as the inorganic core, corroborating that they correspond to rGO and Cu_2_O, respectively.

It is noteworthy that Cu_2_ONPs were not observed when experiments were done using GO-free Cu foil substrates. Therefore, it is quite probably that the Cu-based NPs obtained at 200–1000 °C develop from the preformed copper oxide nanoparticles, which grew during the GO–copper foil sample preparation.

It was reported that the melting process of bulk materials involves a pre-melting stage in which the surface melts at temperatures lower than the bulk material melting point [22–23]. In fact, kinetics studies on metal melting revealed that the pre-melting temperature depends partly on the surface microstructure and partly on surface-adsorbed impurities [24]. It has also been reported that, for nanostructured materials, the melting point strongly depends on the size and it is lower than that of the bulk counterpart [25]. For instance, bulk Cu melts around 1084 °C [26], whereas 40 nm size CuNPs were found to melt at 190 °C, and the surface melting begins around 180 °C [27].

**Figure 3:**
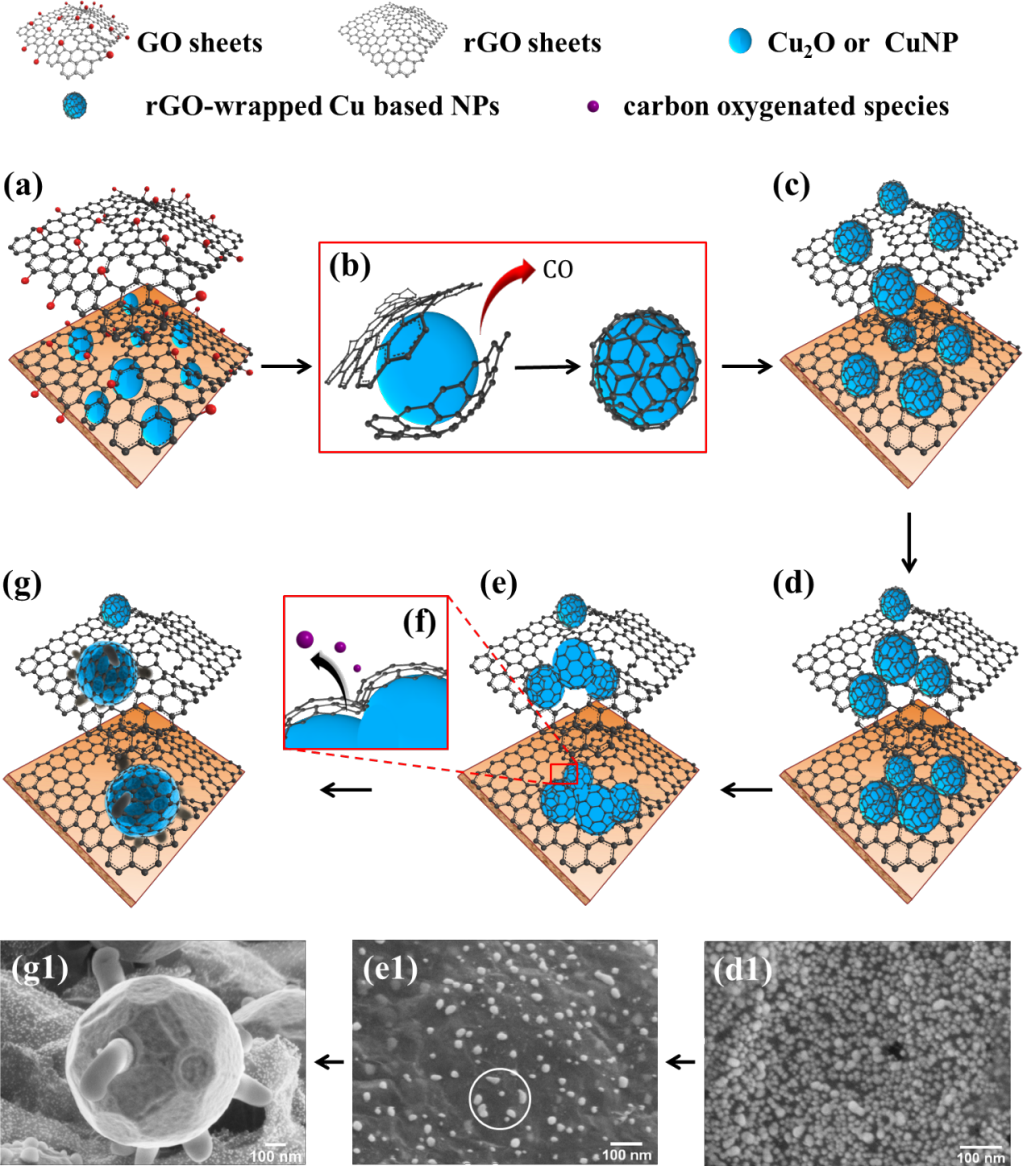
Scheme of the proposed mechanism for the formation of rGO-Cu_2_ONPs and rGO-CuNPs: (a) The Cu_2_ONPs developed during the annealing at 80 °C. Upon raising the temperature from 200 to 1000 °C: (b) reduction of GO sheets (with CO and water vapor emission), and GO wrapping of Cu_2_O (200–600 °C) or Cu (800–1000 °C) NPs, (c) surface diffusion, (d) agglomeration and (e) coalescence of adjacent NPs. (f) At the partially melted Cu surface, rGO sheets self-assemble to develop the rGO coating. (g) Equilibrium crystal shape of representative rGO-CuNPs at 1000 °C. FE-SEM images picturing the steps described above: (d1) agglomeration, (e1), coalescence (circle) and (g1) equilibrium crystal shape. Note that, at annealing temperatures of 800–1000 °C, copper oxide transforms into copper via reduction by CO (Cu_2_O + CO → 2Cu + CO_2_).

As shown in Figure 1a, the copper foil surface displays a fine-grained morphology probably comprising native Cu*_x_*O grains and structural defects that could promote the melting on the Cu foil surface even at 200 °C. On this basis, we assumed that upon raising the temperature, the initial Cu_2_ONPs partially melt, then they interact with the GO network to be finally wrapped in GO sheets. The rGO-Cu_2_ONPs diffuse on the partially melted surface to agglomerate and coalesce and so to form bigger nanostructures. During coalescence, rGO catalytically decomposes producing carbon oxygenated species (epoxide, COOH, C–OH, CO_2_ and CO) and water vapor [28]. Once the nanoparticles attain a certain size, the rGO sheets rearrange at the nanoparticle surface to produce a hermetic rGO coating for Cu_2_O or CuNPs (Figure 3).

It is thought that, at 800–1000 °C, carbonaceous species produced during coalescence could establish carbon supersaturation conditions at the partially melted particle surface, and then to promote the formation of a well-closed conformal coating at the CuNP surface. This might explain the stability against oxidation observed in these particles.

In regarding to dominant phase, we propose that Cu_2_O reacts with gaseous carbonaceous species derived from the GO reduction process to become Cu, this depending on the amount of CO evolved during such a thermal process.

The carbothermal reduction of copper oxide has been studied by several authors, and the involved chemistry and pathway toward the copper oxide reduction appear to be very complex processes [29–31]. Experiments on the reduction of CuO by CO carried out by X. Wang et al. [29] revealed that CuO decomposes either directly to metallic copper when high amounts of CO were supplied or via formation of Cu_2_O when CO supply was limited.

Because the detachment of the oxidative species from the GO plane is a temperature-activated process, we suggest that, in the interval from 200 to 600 °C, CO does not evolve in sufficient amounts to completely reduce the existent copper oxide phase. The resultant nanoparticles increase in size preserving the original phase. In the same view, in the high-temperature range (800–1000 °C), copper oxide was totally converted to copper via reduction by CO.

**Figure 4:**
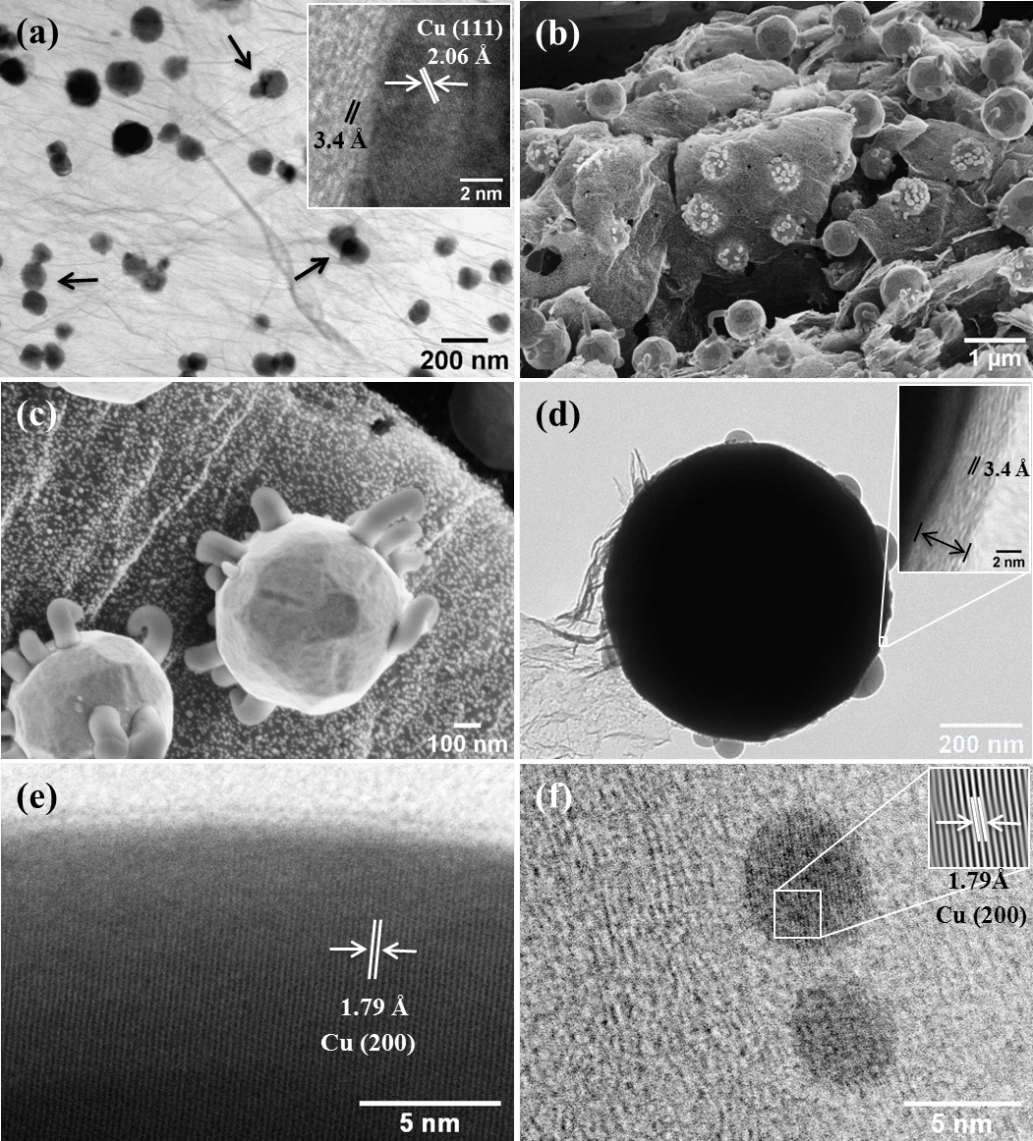
(a) TEM image of a sample annealed at 800 °C for 1 h. (b,c) Low- and high-magnification FE-SEM. (d) TEM images of sample prepared at 1000 °C for 1 h. Inset in (d) provides details of rGO sheets coating the Cu core. (e) The Cu core HR-TEM image and (f) small Cu nanoparticles in the background (see text). Note that, due to the relative large size of Cu nanostructures for TEM observation (Figure 3d and inset), the Cu nanostructure itself and its core–shell feature were separately analyzed.

After being rGO-coated, the NPs attain their equilibrium size and shape depending on the undercooling temperature. Figure 4 displays TEM and HR-TEM images of samples prepared at 800–1000 °C. In this temperature range, elemental Cu develops as the predominant inorganic phase in the core of the rGO-coated nanoparticles. Notice that the Cu_2_O and CuNPs developed a faceted shape, i.e., their equilibrium crystal shape which is determined by the surface energy [32].

### Equilibrium crystal shape development

In all cases, TEM or FE-SEM images revealed that polydisperse rGO-Cu and rGO-Cu_2_ONPs were obtained, some of them displayed a highly faceted morphology. Temperatures lower than 1000 °C resulted in particles 5–170 nm in size with irregular shapes. The considerable dispersion in size and shape can be attributed to nanoparticle coalescence, as indicated by the arrows in Figure 4a.

The FE-SEM images (Figure 4b,c) display the striking morphology of the sample prepared at 1000 °C. It consists of highly faceted core–shell Cu-rGO nanostructures and small, nearly monodisperse 3 nm in size, rGO-CuNPs, all of them decorating the rGO sheets. Some holes in the rGO network are also observed. Previously, we suggested such holes originate from the carbon consumption by the CuNP to get the carbon coating. It is also seen that faceted nanostructures have curved rod-shaped nanostructures attached to specific crystal planes.

As indicated in Figure 4c,d, Cu nanoparticles developed a different faceted Wulff-like structure (or equilibrium crystal shapes [32]). It has been reported that the equilibrium crystal shape for a given material depends partly on the crystalline structure of its bulk counterpart, and partly on the experimental parameters, such as processing temperature, time, and impurity control [33–35]. For bulk Cu, that crystallizes in the fcc structure, its possible equilibrium crystal shapes exhibit the {111}, {100}, {110} and {113} facets, because the Wulff shapes are determined by the lowest energy crystalline planes, as reported by Chatain and co-workers [36].

**Figure 5:**
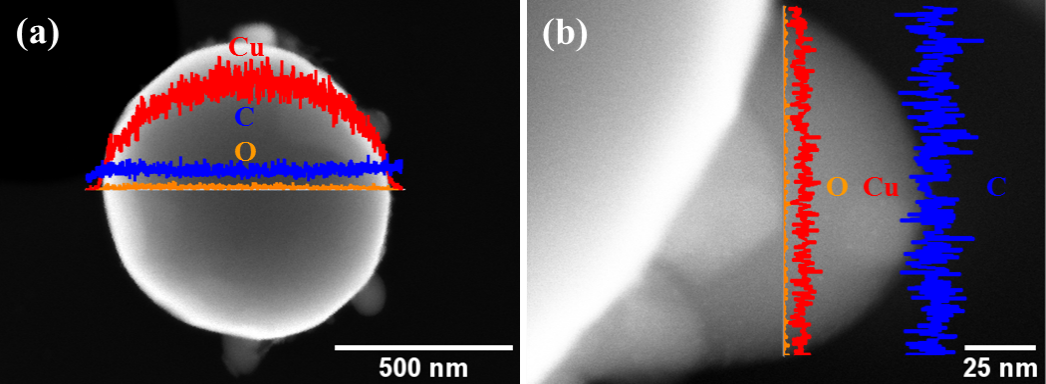
HAAF image and EDS line-scan profile across of (a) single particle showing carbon, copper and oxygen signals, (b) single nanorod showing the carbon, copper and oxygen signals.

We carried out high angle annular dark field (HAADF) measurements to assess the chemical composition of the faceted nanoparticles. HAADF images and EDS line scans of a Cu-rGO nanoparticle and a rod-shaped nanostructure are shown in Figure 5a,b, respectively. From the EDS line-scan analysis in Figure 5a it is seen that the chemical composition of the particle mostly comprises copper and carbon, so corroborating the core–shell structure for faceted Cu-rGO nanoparticle. It is seen that oxygen is also present at low levels because of the partial removal of oxygenated groups from the graphene sheet. An EDS line-scan analysis across a rod-shaped nanostructure (Figure 5b) revealed that it mostly comprised of carbon and traces of copper. We have assumed that the curved rod-shaped nanostructures grow by the catalytic decomposition of GO sheets into carbon and oxygenated species, so promoting carbon supersaturation on the facet surface, and then nucleation and growth.

**Figure 6:**
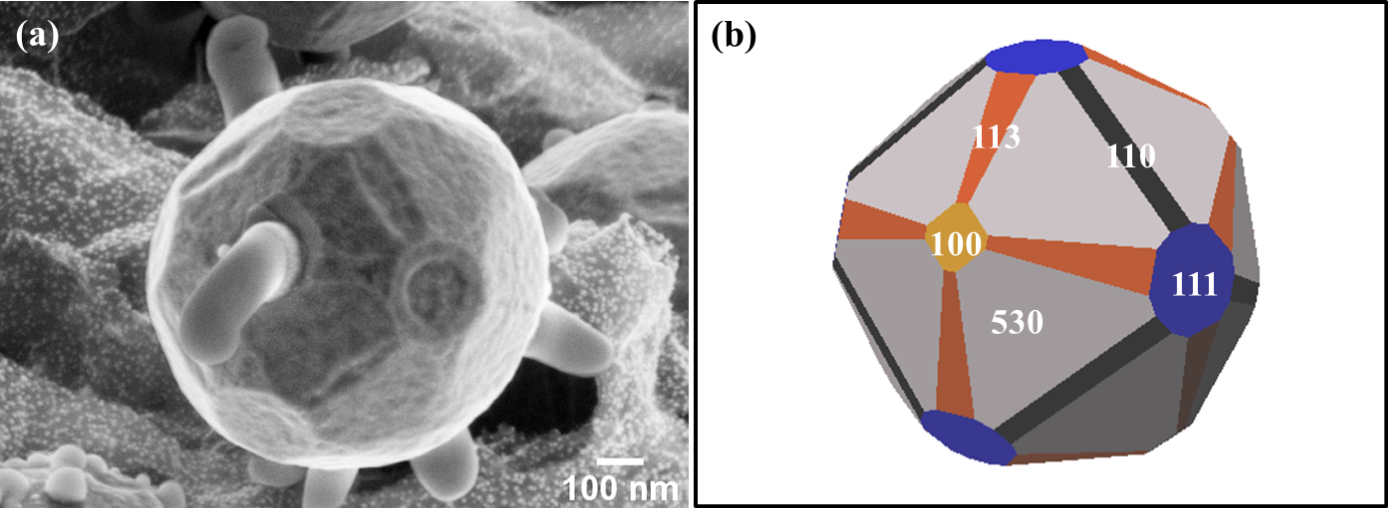
FE-SEM image of (a) single particle showing the different facets (b) schematic crystal shape created using the Wulffman freeware [37–38].

The FE-SEM image (Figure 6) shows the faceted morphology of the core–shell Cu-rGO particle of the sample prepared at 1000 °C, and the corresponding three-dimensional equilibrium crystal shape created using the Wulffman freeware [37–38] (Figure 6b). For the simulation, the crystallographic directions were estimated from the symmetries of the facets [39]. The facets present correspond to {111}, {110}, {100}, {113} and {530}. It is worthy to note that {530} facets are not typically reported for highly pure Cu, but in this case, as explained by Meltzman et al. [40], the presence of carbon layers at the surface, modifies the anisotropy giving rise to the appearance of other facets.

## Conclusion

We describe the formation of faceted core–shell Cu_2_O-rGO and Cu-rGO nanoparticles on the surface of a Cu foil by annealing foil-supported graphene oxide (GO) under an Ar atmosphere. The predominant phase as well as the particle size and shape strongly depend on the process temperature, in the ranges of 200–600 °C and 800–1000 °C, Cu_2_O or Cu were obtained, respectively. It was demonstrated that rGO plays a dual role of stabilizing and protecting the Cu nanoparticles from further oxidation. In particular, at 1000 °C, the Cu nanoparticles exhibit an equilibrium crystal shape. The facets found out by simulation were {111}, {110}, {100}, {311} and {530}. The faceted nanoparticles display arm-like carbon nanorods originating from different facets of the copper crystal structure.
